# Multiplexed biomimetic lipid membranes on graphene by dip-pen nanolithography

**DOI:** 10.1038/ncomms3591

**Published:** 2013-10-10

**Authors:** Michael Hirtz, Antonios Oikonomou, Thanasis Georgiou, Harald Fuchs, Aravind Vijayaraghavan

**Affiliations:** 1 Institute of Nanotechnology (INT) and Karlsruhe Nano Micro Facility (KNMF), Karlsruhe Institute of Technology (KIT), Hermann-von-Helmholtz-Platz 1, 76344 Eggenstein-Leopoldshafen, Germany; 2 School of Computer Science and Centre for Mesoscience and Nanotechnology, The University of Manchester, Manchester M13 9PL, UK; 3 School of Physics and Astronomy, The University of Manchester, Manchester M13 9PL, UK; 4 Physical Institute and Center for Nanotechnology (CeNTech), University of Münster, Wilhelm-Klemm-Str. 10, 48149 Münster, Germany; 5 School of Materials and Centre for Mesoscience and Nanotechnology, The University of Manchester, Manchester M13 9PL, UK; 6 These authors contributed equally to this work

## Abstract

The application of graphene in sensor devices depends on the ability to appropriately functionalize the pristine graphene. Here we show the direct writing of tailored phospholipid membranes on graphene using dip-pen nanolithography. Phospholipids exhibit higher mobility on graphene compared with the commonly used silicon dioxide substrate, leading to well-spread uniform membranes. Dip-pen nanolithography allows for multiplexed assembly of phospholipid membranes of different functionalities in close proximity to each other. The membranes are stable in aqueous environments and we observe electronic doping of graphene by charged phospholipids. On the basis of these results, we propose phospholipid membranes as a route for non-covalent immobilization of various functional groups on graphene for applications in biosensing and biocatalysis. As a proof of principle, we demonstrate the specific binding of streptavidin to biotin-functionalized membranes. The combination of atomic force microscopy and binding experiments yields a consistent model for the layer organization within phospholipid stacks on graphene.

## Abstract (web summary)

The sensitivity and selectivity of graphene-based biosensors depends on attaching various functional groups to graphene. Hirtz *et al*. use dip-pen nanolithography to directly write phospholipid membranes on graphene, which enables multiplexed and heterogeneous non-covalent functionalization.

## Graphical abstract

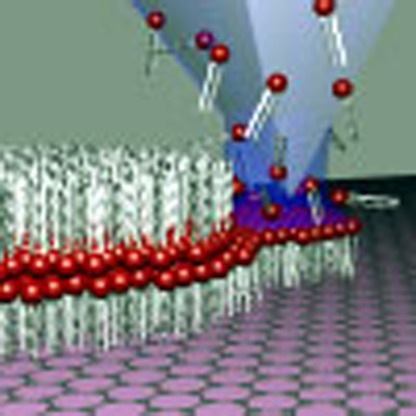

Graphene in its pristine state and various functionalized derivatives has yielded excellent results in sensing experiments, such as single-atom gas sensitivity^1^, DNA sequencing^2^,^3^,^4^ and electronic nose^5^. Graphene sensors can operate in a number of different modalities^6^, such as electronic, electrochemical, optoelectronic and nano-electromechanical sensing. There have been a number of demonstration of graphene-based sensors; however, the high sensitivity and selectivity needs to be engineered by attaching various functional groups to graphene, either covalently^7^ or non-covalently^8^. In applications in biosensing, a number of receptor molecules that could impart selectivity to graphene would work best as intended if they existed in their native environment, for instance, in a phospholipid cell membrane. In many cases, direct covalent or non-covalent binding of functional groups on graphene can often suppress their behaviour or even denature them^9^. When interfacing graphene with biological systems such as cells^10^ or in using it as transducer for biological sensing, an overlay on graphene mimicking cellular membranes could provide a more native environment. One option to achieve this is the assembly of biomimetic lipid membranes on graphene, which was recently demonstrated by vesicle fusion^11^,^12^. Other self-assembled monolayers have been deposited on graphene using a variety of techniques^13^. However, these methods lack the ability for a direct spatial control on membrane formation and cannot deposit membranes of different compositions on one device. A functional multiplexed graphene-based sensor, such as an electronic nose or tongue, could be achieved with lipid membranes localized over a specific graphene region as well as membranes of different composition adjacent to each other, with microscale precision^1^,^14^.

Dip-pen nanolithography^15^ (DPN) has been used for molecular deposition on graphene^16^,^17^ and to pattern a variety of biological active substances onto other solid supports^18^. DPN with phospholipids (L-DPN) enables the decoration of solid supports with tailored patches of lipid membrane that can be used as *in vitro* membrane model^19^. The technique was already utilized in a range of applications from allergen presentation^20^,^21^ to sensor functionalization^22^,^23^,^24^. In addition to allowing for spatial control of the fabricated membrane patches, L-DPN also offers multiplexing^20^ (that is, parallel deposition of different ink compositions), therefore effectively addressing the challenge of incorporation of different membrane compositions within one target area.

Here we present our results towards the fabrication of lipid membrane patches onto graphene using L-DPN, demonstrating the precise and selective spatial positioning and shaping of the pattern, enabling multiplexed and localized heterogeneous functionalization. Moreover, it is a direct writing method that does not require masks or other protective layers, therefore minimizes unwanted exposure of the graphene to additional chemicals and processing steps.

## Results

### L-DPN on graphene

**Figure 1:**
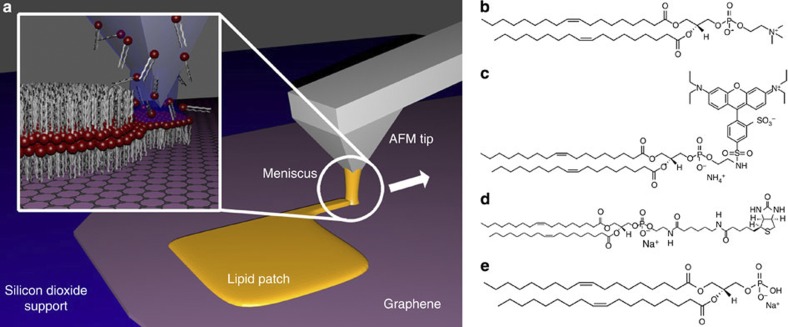
Schematics of DPN and lipid molecule structures. (**a**) Scheme of the writing process and (**b**–**e**) the phospholipids used in our study: (**b**) the main constituent of the phospholipid ink used as carrier, DOPC and three functional admixtures (**c**) the fluorescently labelled Liss Rhod PE, (**d**) the biotinylated Biotinyl Cap PE and (**e**) the negatively charged DOPA.

The schematic writing process and molecular structure of lipids used in this work are shown in Fig. 1. In L-DPN, an atomic force microscopy (AFM) tip covered with the desired lipid mixture is brought into contact with the target area on the substrate and then steered over the surface in the desired pattern (Fig. 1a). The lipids transfer over a water meniscus formed under the controlled environmental humidity of the system from the tip to the substrate and self-assemble into membranes. 1,2-dioleoyl-sn-glycero-3-phosphocholine (DOPC, Fig. 1b) was used as the main component of the phospholipid inks used in L-DPN functionalization of the graphene. Because of the preferable writing behaviour, DOPC is used as a carrier to facilitate the writing of lipids that could not be used for L-DPN in pure form^19^. To this carrier, 1 mol% of 1,2-dioleoyl-sn-glycero-3-phosphoethanolamine-*N*-(lissamine rhodamine B sulfonyl) (Liss Rhod PE, Fig. 1c), 5 mol% of either 1,2-dioleoyl-sn-glycero-3-phosphoethanolamine-*N*-(cap biotinyl) (Biotinyl Cap PE, Fig. 1d) or 1,2-dioleoyl-sn-glycero-3-phosphate (DOPA, Fig. 1e) were added, yielding three different functional ink mixtures. The first lipid mixture being fluorescent (abbreviated Rhod-PE in the following) gives an easy detectable marker for optical control of the lithographic outcome, at least in case of writing on silicon dioxide. The second lipid mixture (abbreviated Biotin-PE in the following) can act as a model for active sensor elements by its high affinity to binding streptavidin^25^, a concept widely used in biotechnology for the linking and immobilization of various proteins and other bioactive compounds. The third lipid mixture (with DOPA) was used to probe the effect of negative charge doping on the graphene. After the preparation of inks and application of the inks to one-dimensional cantilever arrays (see Methods section for details), the lipid membranes were written onto graphene flakes and neighbouring areas of silicon dioxide for comparison. To elucidate the feasibility of using the physisorbed lipid patches in liquid environments, which would be necessary for application in biosensing and cell interfacing, binding experiments with fluorescently labelled streptavidin were conducted.

**Figure 2:**
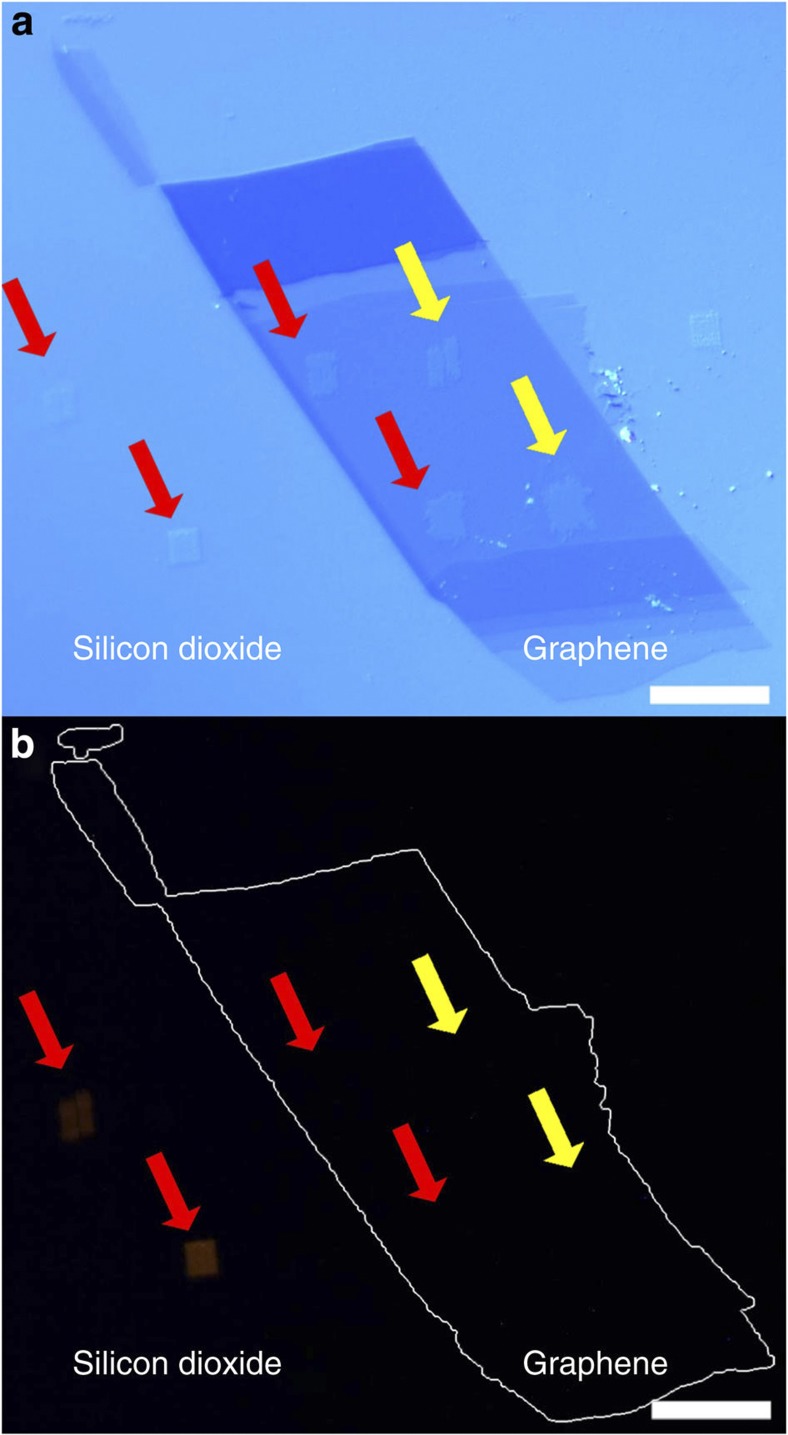
Optical imaging of lipids on graphene. (**a**) Optical microscopy image of graphene sheets on silicon dioxide support after functionalization with lipid membrane patches. Positions of patches are marked by arrows (red for Rhodamine-PE, yellow for Biotin-PE) and (**b**) corresponding fluorescent image. Fluorescence of Rhodamine-PE patches on the graphene is quenched. The outline of the graphene sheet is marked in **b** for reference. Scale bars, 20 μm.

Figure 2 shows a representative outcome of the lithographic process with several 5 × 5 μm^2^ lipid patches written to the graphene and some neighbouring areas of silicon dioxide (images of the writing process are given in Supplementary Figs S1 and S2). In Fig. 2, the different patches are marked for their composition with arrows, red indicating patches of Rhod-PE and yellow Biotin-PE. By comparing the optical micrograph (Fig. 2a) with a fluorescent image (Fig. 2b), it becomes obvious that the bright orange fluorescence of the Liss Rhod PE that can be observed in the patches on the silicon dioxide is quenched completely by the underlying graphene. This indicates a conformal and thin lipid layer in intimate contact with the graphene substrate, allowing for an efficient charge transfer interaction between the fluorophore and graphene.

### Atomic force microscopy studies

**Figure 3:**
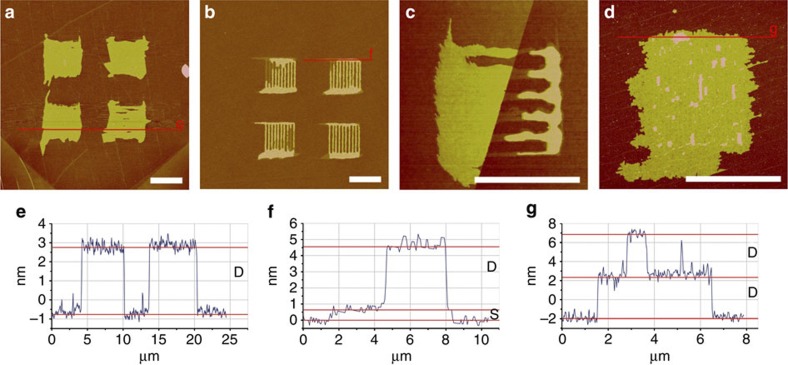
AFM images of phospholipid membranes on graphene and silicon dioxide. (**a**) Four 5 × 5 μm^2^ phospholipid patches on graphene. (**b**) Patches written under the same writing conditions as **a** but on neighbouring silicon dioxide area. (**c**) Close up of a patch written directly on the edge of a graphene flake (left) and (**d**) image of a membrane written onto graphene with some residues of the exfoliation process present on the graphene before the lipid DPN process. (**e**–**g**) show the line profiles indicated in **a**, **b** and **d**, respectively. The different observed height levels D and S are indicated in the line profiles. Scale bars, 5 μm.

AFM imaging in air reveals that the phospholipids form flat uniform layers on the graphene (Fig. 3a,d). Applying the same writing parameters (30% relative humidity (R.H.), 500 nm pitch of hatch lines) on the silicon dioxide leads to non-merged lipid patches; the hatch lines used to fill the square area are still distinctly visible (Fig. 3b). To obtain a merged membrane on the silicon dioxide, either the humidity at writing must be raised or the pitch distance of the hatch lines reduced. For a given set of writing parameters, the lipids assemble preferentially on graphene compared with silicon dioxide. This becomes evident from the observation that when a lipid patch is written across a graphene—silicon dioxide interface, a disproportionately large quantity of the lipid is deposited onto the graphene surface to assemble there, indicating a significantly higher tip-substrate transfer rate for the lipids onto the graphene compared with silicon dioxide (see Supplementary Fig. S3). The reason for the enhanced transfer rate is likely to be the strong interaction between the phospholipids hydrocarbon tails and the graphene^26^. Another difference between the patches on the silicon dioxide in comparison with the ones on graphene is the occurrence of two distinct height levels in the patches instead of only one as it is in the case on graphene. Figure 3c shows a patch that is directly written over the edge of a graphene flake (left in the image), making the difference in spreading obvious. The uniform height level of the lipid patch on the graphene part should also be noted in contrast to the two distinct height levels on the silicon dioxide part in this image. Two different types of height steps can generally be observed (Fig. 3e–g): one thin-layer step of about 1 nm thickness (marked as S in the profile lines) and one thicker of about 4 nm (marked as D). The averaged thickness of the D-layer in Fig. 3a is (3.7±0.2) nm and (4.0±0.2) nm in Fig. 3b,d; the S-layer in Fig. 3b is (0.9±0.2) nm. The phospholipid patches tend to spread out to a completely one-level layer on the graphene, indicating a higher mobility as compared with the silicon dioxide. The only occasion where multilayers were observed on graphene is when residues of the exfoliation process are present on the graphene surface presumably hindering complete spreading and therefore allowing stacking of the membrane (Fig. 3d). Comparing the layer structures on silicon dioxide and graphene, it becomes clear that S-layers are only observed on silicon substrates and are always the base layer, never seen on top of another layer. Layers on top of this base layer are always D-layers. In contrast, on graphene only D-layers are observed, as base layer and as added layers in case of hindered spreading by polymer residue or wrinkles in the graphene arising from the exfoliation process (Fig. 3g).

### Spreading studies of lipids on graphene

**Figure 4:**
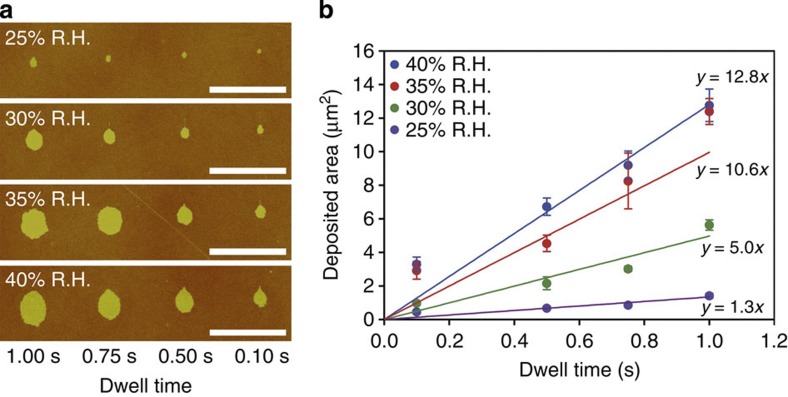
Lipid transfer for different dwell times and humidity. (**a**) Dot features written by bringing an AFM tip inked with Rhodamine-PE into contact with the graphene substrate for different amounts of time and at different humidity (R.H.). Scale bars, 10 μm. (**b**) Graph of the dependence of dot area on dwell time for different humidity. Error bars are given as one s.d. of four points averaged per data point. The linear fits yield diffusion constants of 1.3–12.8 μm^2^ s^−1^ with rising humidity.

To establish the dependence of lipid transfer and feature size on writing speed and humidity, dot patterns with different tip-substrate contact time were written at different humidity (Fig. 4a). The transfer characteristics for lipids to graphene substrates observed in our experiments follow the diffusion models established for the transport of thiols on gold by DPN^27^. The deposited area of lipid membrane in a dot feature is rising for longer dwell times as well as for higher humidity (Fig. 4b). Linear fits to the obtained data yield increasing transfer constants of 1.3–12.8 μm^2^ s^−1^ while increasing humidity from 25–40% R.H. The transfer constants derived by linear fits to the data are tabulated in Supplementary Table S1. At a low dwell time of 0.1 s (lowest achievable in our set-up), the deposited dot area is higher than expected from the diffusion model, which can be assumed to be caused by the small amount of lipid that is expected to be deposited by adhesion alone on touching. Similar tests for line features written at different writing speeds and humidity yielded line widths varying from 130 nm to 1.4 μm. A full overview over of the dot and line patterns written with varying dwell time and writing speed is given in Supplementary Fig. S4. To assess the writing of lines, a pattern of 10 μm long lines with 5 μm pitch was written at different humidity (Supplementary Fig. S4b). The broadening at the starting point of the line (all written from bottom of the images to the top) is caused by the DPN set-up pausing for a short time after contacting the surface and before starting to write the line. During this time, lipid is already transferred to the substrate surface. The line width of each line was measured at five different positions along the line, avoiding the broadened parts at the beginning. Depending on the humidity, the lines written with highest speed were partly discontinuous (30 and 20 μm s^−1^ for 25% R.H. and 30 μm s^−1^ for 30% R.H.). The line features are increasing in width with increasing humidity and decreasing writing speed (Supplementary Fig. S4d). The overall characteristics are compatible with diffusion models established for the writing of lines with thiols on gold, the breaking up of the lines for combinations of low humidity and high writing speed being attributed to a lack in ink reflow to the tip apex^28^. This phenomenon also explains why at lower humidity the writing speed needs to be lowered more to reach continuous lines. Comparing the transfer constants in the lithography of dot features with that of the line patterns, it becomes obvious that both scale roughly linear with humidity but the transfer rates when writing lines are generally lower while starting from similar values for low humidity of 25% R.H. (Supplementary Fig. S5). This underlines the notion that writing of dot and line features must be described with different transfer parameters because of the different geometry and the constant exposure to pristine (not covered by ink) substrate area while writing lines.

### Raman spectroscopy of lipid-coated graphene

**Figure 5:**
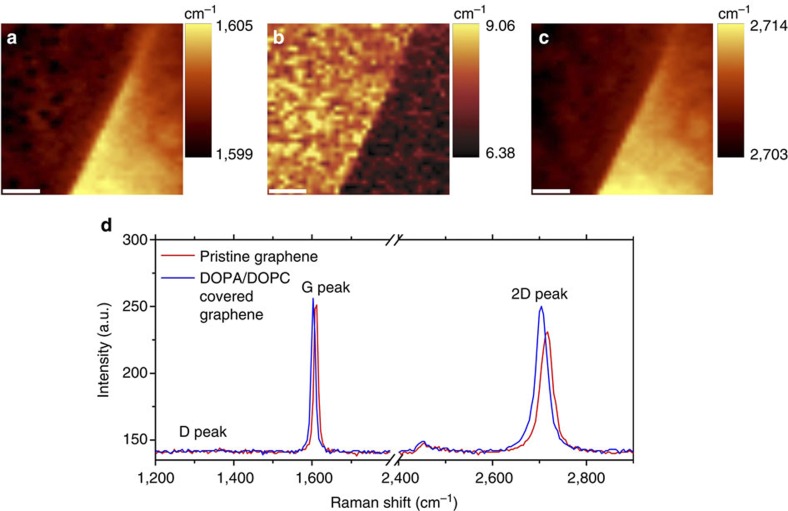
Raman spectroscopy of lipids on graphene. (**a**–**c**) Raman maps across the boundary of a lipid membrane, showing lipid-covered graphene on the left half and pristine graphene on the right half. (**a**) G-peak position, (**b**) G-peak full width at half maximum, (**c**) 2D-peak position. Scale bar, 2 μm (**a**–**c**). (**d**) Representative Raman spectra from lipid-covered and pristine graphene regions showing the downshift and narrowing of G peak and of 2D peak arising from interaction with the *n*-doping lipid.

The charge transfer interaction between lipids and graphene and the integrity of the graphene during the writing process were confirmed by Raman spectroscopy. Figure 5 shows the Raman maps and representative spectra of graphene covered with DOPA/DOPC, as well as adjacent uncovered graphene. The pristine graphene is heavily *p*-doped because of the underlying silicon dioxide substrate^29^. This is reflected in the Raman spectrum as a highly upshifted G and 2D peak positions as well as a very narrow full width at half maximum of the G peak^30^. The peak positions of undoped graphene for the 488-nm excitation are 1,583 cm^−1^ (G) and 2,691 cm^−1^ (2D)^31^. When the graphene is covered by the lipid membrane, the G and 2D peaks downshift closer to the charge neutrality point (become less *p*-doped), the G peak broadens and the 2D to G intensity ratio increases, all of which are consistent with electron transfer from the *n*-doping DOPA in the lipid membrane. This can be distinguished from *p*-doping, where the 2D peak will further upshift rather than downshifting. In addition, no defect related D peak is observed in the graphene before and after the L-DPN writing, indicating that the lithography process does not damage the graphene.

### Streptavidin binding on biotinated lipids

Having established that (1) lipids form uniform membranes on graphene, (2) lipid membranes can be assembled on graphene in a multiplexed fashion in close proximity, and (3) there is a charge transfer interaction between the lipid membranes and graphene, we propose that lipid membranes can be used as a non-covalent approach to present functional groups on a graphene surface for applications such as biosensors, biocatalysis and interfacing with living cells. As a demonstration for biosensing capabilities and membrane stability, we performed streptavidin-binding experiments on biotinylated lipid membranes assembled on graphene.

**Figure 6:**
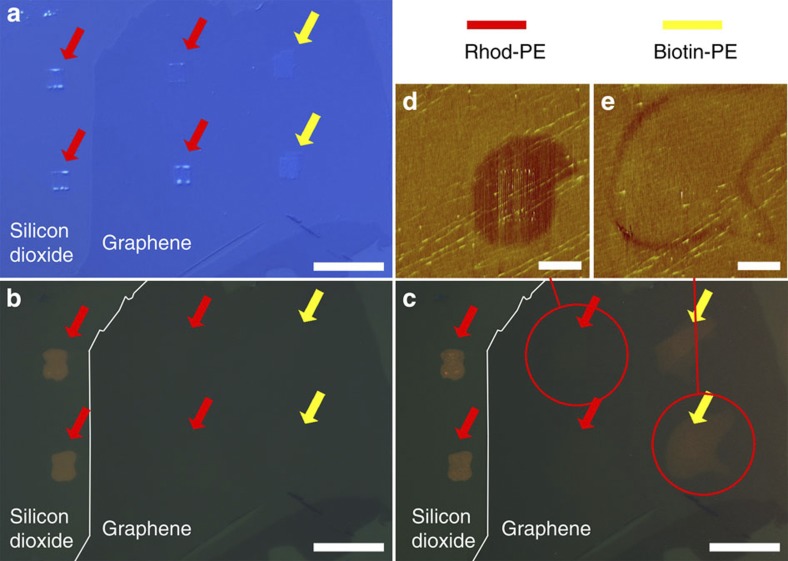
Binding of fluorescently labelled streptavidin to the functionalized graphene. (**a**) Optical image of sample after application of the lipid patches (in air); positions of patches are marked by arrows (red for Rhodamine-PE, yellow for Biotin-PE). (**b**) Sample after immersion into buffer as a combined optical and fluorescence image. Rhodamine-PE patches are visible in fluorescence on the silicon dioxide area, but quenched on graphene. (**c**) Combined optical/fluorescence image after incubation of the sample with fluorescently labelled streptavidin. Rhodamine-PE patches are still quenched, but Biotin-PE patches become visible in fluorescence now. AFM images obtained in liquid after incubation with streptavidin are given in **d** for a Rhodamine-PE patch and (**e**) for a Biotin-PE patch, area of images indicated by red circles in **c**. The left border of the functionalized graphene sheet is outlined in images **b** and **c**. Scale bars, 20 μm (**a**–**c**); 5 μm (**d**,**e**).

A typical outcome of binding experiments with streptavidin is shown in Fig. 6. Figure 6a shows a graphene sample decorated with patches of Rhod-PE and Biotin-PE as described previously. Figure 6b shows the same sample after immersion into PBS buffer in a combined optical and fluorescent image. The fluorescent Rhod-PE patches are clearly visible on the silicon dioxide area next to the graphene flake, whereas the corresponding patches on the graphene are still quenched under liquid. After blocking the non-lipid-functionalized areas of the substrate with BSA dissolved in PBS, the sample was incubated with a solution of fluorescently labelled streptavidin. By covering all surface areas prone to protein adsorption that are not already protected by the phospholipid functionalization, the BSA prevents nonspecific binding of the fluorescently labelled streptavidin^32^, allowing for unambiguous detection of only the specific adsorption to its biotin target. Now, the Biotin-PE patches light up in the fluorescence image (Fig. 6c), indicating successful selective binding of the streptavidin to the graphene-supported lipid membrane. Neither the silicon dioxide nor the graphene areas show significant background fluorescence, implying equally good blocking against nonspecific binding of the streptavidin by the BSA on both substrates. The size of the streptavidin is obviously large enough to put the attached fluorophore far enough from the graphene surface to prevent (total) quenching of the fluorescence in this case. In comparison with the lipid patches on the silicon dioxide, the patches on the graphene spread over a much bigger area of the surface, again indicating a higher mobility. AFM in liquid was employed to scan these areas after incubation with streptavidin. The Rhod-PE patch is showing a negative height contrast of (−1.1±0.1) nm averaged, caused by the lipid layer being thinner than the surrounding BSA layer introduced in the blocking step of the streptavidin incubation to prevent nonspecific binding on the exposed graphene and silicon dioxide areas (Fig. 6d). The streptavidin attaching to the biotinylated phospholipid in the Biotin-PE patch fills up this height difference; therefore, the Biotin-PE layer shows almost no height difference and can be only discerned from the surrounding BSA layer by a small height depression at the outline of the patch (Fig. 6e). It can also be inferred that the membrane spreading has occurred rapidly on exposure to the aqueous medium before the BSA blocking layer is formed in the surrounding areas.

### The structure of lipid membranes on graphene

The results presented above describe the controlled delivery and subsequent self-assembly of phospholipid membranes on graphene and shed light on the organization of the obtained artificial biomembranes, as will be discussed in the following. As described above, only D-layers were observed on graphene, whereas patches on silicon dioxide always consist of a base S-layer with additional D-layers on top. This agrees with previous studies of lipid patches on silicon dioxide that show additional layers on top of the thin base layer are always of same thickness—the interpretation being that the thin base layer is a phospholipid monolayer, facing headgroups towards the hydrophilic substrate, decorated by stacks of phospholipid bilayers with the hydrocarbon chains facing outwards^33^. The phospholipid monolayers can be identified with the S-layer and the double layers with the D-layers discussed in the current work. As graphene is a hydrophobic substrate^34^,^35^, this model can be neatly extended towards the observation of only one type of step height on graphene.

**Figure 7:**
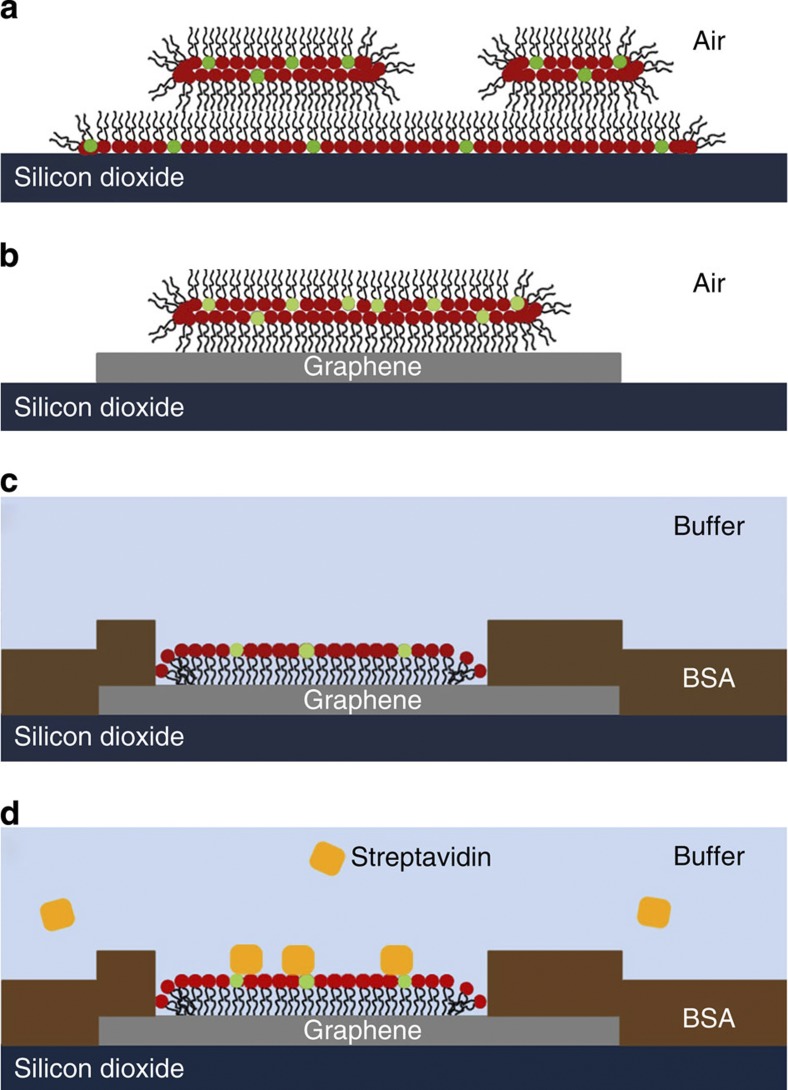
Proposed membrane organization on silicon dioxide and graphene. DOPC headgroups are marked in red, Biotin-PE headgroups in green. (**a**) Base monolayer and additional bilayer on silicon dioxide in air, (**b**) single bilayer on graphene in air and (**c**) monolayer of phospholipids on graphene surrounded by BSA layer under water. Streptavidin can later be bound to the biotinylated headgroups of the phospholipids from solution (**d**) with BSA and DOPC preventing unspecific binding to the substrate.

The situation on the hydrophilic silicon dioxide substrate is depicted in Fig. 7a: the thin base S-layer consisting of a phospholipid monolayer is partly covered with a second D-layer consisting of a phospholipid bilayer, yielding two distinct height steps. With the hydrophobic graphene, there cannot be a base S-layer with hydrophilic headgroups facing towards the substrate; thus, only one height step can be observed that of a D-layer in form of a phospholipid bilayer (Fig. 7b). The lower thickness of the S-layers (significantly less than half of a D-layer) indicates a less dense packing of the phospholipids in this leaflet-type membrane compared with the full bilayer, allowing more entanglement of the hydrocarbon chains, yielding a lower thickness. On immersion into liquid, the patches have to reorganize, now exposing the hydrophilic headgroups as terminating interface to the liquid. This could either happen by shedding the outer leaflet of the double layer or by spreading of the outer leaflet onto the graphene substrate. Taking into account the strong interaction between the hydrocarbon chains of the phospholipids and the hydrophobic graphene^26^ and the observed extensive spreading in comparison with the silicon dioxide substrate, we infer that the latter is happening in our experiment. We also report a different membrane organization compared with those inferred previously by vesicle fusion on graphene^11^ and graphene oxide^12^. Vesicle fusion, where the sample is already immersed in buffer solution during membrane formation, yields a phospholipid bilayer on the graphene with the headgroups facing outwards separated from the graphene by a thin interfacing water film. In contrast, L-DPN yields an inverted phospholipid bilayer on graphene in air with the headgroups inwards that reorganizes into a monolayer on immersion into aqueous media. The BSA blocking fills up the graphene and silicon dioxide areas not covered by phospholipids, leading to a situation depicted in Fig. 7c, explaining the negative height contrast in AFM under liquid. On the incubation with streptavidin in solution, the streptavidin binds selectively to the biotin headgroups of the Biotinyl Cap PE (Fig. 7d). The streptavidin binding to the Biotin-PE patches will then fill up the height difference towards the surrounding BSA layer. It should be noted that whereas the graphene itself is prone to nonspecific adsorption in the same way than the silicon dioxide substrate, therefore needing BSA blocking, the phospholipid patches themselves counter nonspecific binding. Only the biotinylated lipids in the Biotin-PE patches allow for binding of the streptavidin, whereas the Rhod-PE patches in lack of specific binding motives repel streptavidin. Therefore, functionalization of graphene by phospholipid membranes provides safeguard against nonspecific binding at the same time. The quenching of the Liss Rhod PE fluorescence in contrast to the retained fluorescence of the bound streptavidin-cy3 demonstrates that the thickness of the membranes is in the relevant regime for tuned quenching effects. This could be exploited in fluorescence-quenching assays likewise to the detection of DNA hybridization on graphene by restored fluorescence^36^.

## Discussion

L-DPN has previously been demonstrated on a variety of substrates such as silicon dioxide, titanium and polystyrene^19^. Graphene, however, is an important new substrate for biomimetic lipid membrane assembly for a number of reasons. The electronic charge transfer that is possible between lipids and graphene will allow for fluorescence quenching-based assays, which are not possible with insulating substrates. The extent of quenching can be controlled by the distance of the fluorophore from the graphene^37^. The carrier density in graphene, which is a semi-metal, can be modulated through this charge transfer interaction, which we observed here through Raman spectroscopy. This modulation of carrier density can lead to electronic sensors based on the graphene-lipid system. Such carrier density modulations cannot occur on other metallic substrates such as gold. Graphene has been proposed as an electrode material to interface with live cells, and a graphene-supported lipid membrane might be the ideal system for such an interface, which in turn will allow us to electronically address cells or stimulate various biological processes^10^.

In summary, we have demonstrated the assembly of active lipid membranes on graphene of controlled location and size with submicron resolution. The membrane organization can be understood in terms of hydrophilicity differences between silicon dioxide and graphene. On the graphene, inverted phospholipid bilayers (hydrocarbon chains out) are formed in air that reorganize into single layers (hydrocarbon chains facing to the graphene) on immersion into buffer solution. Although the phospholipids are found to be more mobile on the graphene substrate, allowing for uniform spreading, the formed membranes are still stable enough to allow for immersion into liquid and binding of analytes from solution. More importantly, it was demonstrated that multiplexing of different membrane compositions in close proximity can be achieved on graphene by L-DPN, enabling the crucial control over spatial distribution and chemical composition lacking in self-assembled membranes yielded by vesicle fusion. On the basis of the current results and taking into account appropriate measures to control spreading in immersion, for example, by tailoring graphene devices with distinct graphene stripes or applying chemical barriers in forms of polymers or photoresist, L-DPN-generated membranes could become active elements in graphene-based sensor devices and graphene-based biointerfaces with cells.

## Methods

### Preparation of graphene substrates

The graphene flakes used in this work were prepared by micromechanical cleavage^38^,^39^ of natural graphite at the surface of oxidized Si wafers and at the surface of non-reflective, contrast-enhancing sample substrates that are called SURFs (Nanolane, France), whose surface layer is also silicon dioxide. The number of layers of the fabricated flakes was confirmed by a combination of optical contrast^40^,^41^, Raman spectroscopy^42^ and AFM measurements^38^.

### Preparation of phospholipid mixtures

All phospholipids used in our experiments were obtained dissolved in chloroform from Avanti Polar Lipids, USA and used as received. The 20 mg ml^−1^ carrier solution of DOPC was admixed with appropriate amounts of either the fluorescently labelled Liss Rhod PE to obtain a 1-mol% concentration of Liss Rhod PE in DOPC, or the biotinylated Biotinyl Cap PE or the negatively charged DOPA to obtain a 5-mol% concentration of Biotinyl Cap PE or DOPA in DOPC, respectively.

### Lithographic set-up

All writing processes were performed on an NLP2000 system (NanoInk, USA) with M-type one-dimensional cantilever arrays (NanoInk). Matching inkwells (NanoInk) were loaded with 1.5 μl of the different phospholipid mixtures, and then dried in a vacuum desiccator for 15 min. The cantilever array was inked in the inkwells for 10 min at a humidity of 70% R.H. After inking, excess ink was wiped off by writing on a sacrificial area on the sample. Lithography on graphene was typically performed at humidities of 20–30% R.H. with a line pitch of 500 nm and a writing speed of 5–10 μm s^−1^.

### Streptavidin binding

For the streptavidin-binding experiments, the sample was first covered with 100 μl PBS and imaged optically for characterization (spilling was prevented by covering the sample edges with a ring of parafilm (Pechiney Plastic Packaging, USA)). After this, PBS was replaced with 50 μl of a solution of 5% BSA in PBS and allowed to rest for 10 min. Subsequently, the BSA solution was removed and the sample was washed three times by pipetting 50 μl PBS on and off for three times each. Then the sample was incubated for 10 min with a solution of 1:100 streptavidin-cy3 (Sigma-Aldrich, Germany) in PBS. Before fluorescent imaging, the sample was again washed three times with 50 μl PBS (each time pipetting on and off for three times) and covered with 100 μl PBS in the end. All procedures were done at room temperature.

### Imaging set-up

Surface enhanced ellipsometric contrast microscopy^43^,^44^ and fluorescence microscopy images were obtained on a Zeiss Axio Imager set-up equipped with SARFUS Software (Nanolane). AFM images were obtained on a Dimension Icon system (Bruker, Germany). Measurements in air were done in tapping mode with Tap300Al-G cantilevers (BudgetSensors, Bulgaria) with 300 kHz nominal resonance frequency. Measurements in liquid were done in tapping mode with SNL-10 cantilevers (Bruker). The cantilever D with a nominal resonance frequency of 18 kHz was used. Raman spectra and maps were measured on a WiTEC confocal spectrometer with a 488-nm laser, a × 50 objective and an 1,800 lines per mm grating (spectral resolution of 3 cm^−1^), with a laser power well <1 mW to avoid sample damage. The peaks were fitted with a single Lorenzian line shape to determine peak position and full width at half maximum. The areas of the dot features for determining the transfer constants were measured with ImageJ^45^.

## Author contributions

M.H. and A.V. conceived the research and designed the experiments. A.O. fabricated the graphene substrates. M.H. performed the functionalization, AFM and optical characterization and binding experiments, and T.G. and A.O. performed Raman spectroscopy. M.H. and A.V. analysed and interpreted the results, in discussion with H.F. All authors contributed to writing the manuscript.

## Additional information

**How to cite this article:** Hirtz, M. *et al*. Multiplexed biomimetic lipid membranes on graphene by dip-pen nanolithography. *Nat. Commun.* 4:2591 doi: 10.1038/ncomms3591 (2013).

## Supplementary Material

Supplementary Information Supplementary Figures S1-S5 and Supplementary Table S1
