# Supplementary material for: Multiplexed biomimetic lipid membranes on graphene by dip-pen nanolithography
Source: Nat Commun. 2013 Oct 10;4:2591. doi: 10.1038/ncomms3591 (PMC3826641; doi:10.1038/ncomms3591)
Supplement: Supplementary Information — Supplementary Figures S1-S5 and Supplementary Table S1 [file ncomms3591-s1.pdf]

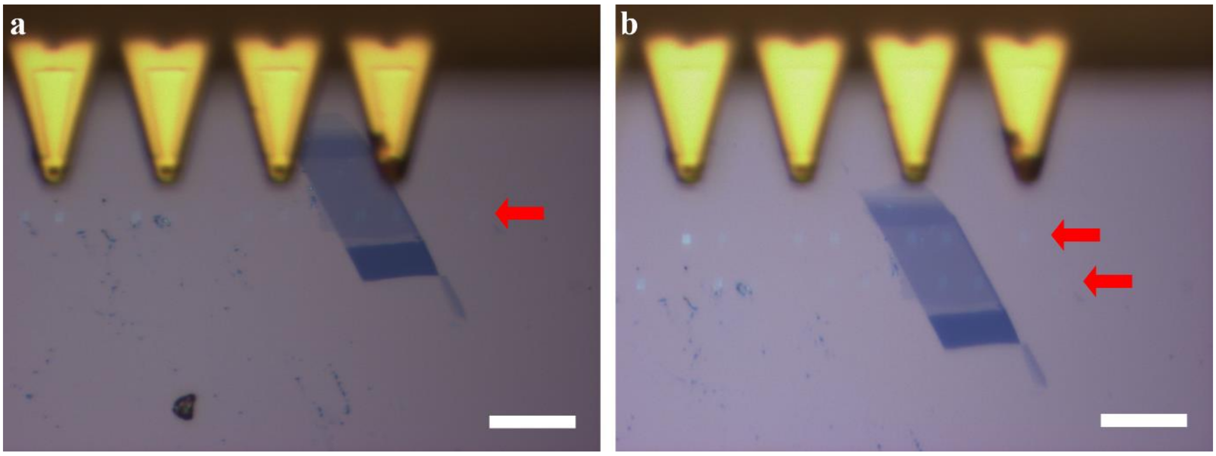

Supplementary Figure S1 | Multiplexed writing process. The 4 rightmost cantilevers of a 12 cantilever array (pitch 66 µm) are visible in top views of a graphene substrate during the writing process. Image (a) shows the situation directly after the first lithography run, the written row of lipid patches indicated by the red arrow. First, the cantilevers write one patch each, then the array is shifted by 45 µm to the right and a second patch per cantilever is written. This enables to bring the patches written by different cantilevers nearer to each other than the 66 µm pitch of the array. The image depicts the situation after the arrays is shifted back left by 45 µm and up by 20 µm. The rightmost cantilever is aligning up in horizontal position with the first patch on the graphene written by this cantilever. Image (b) was taken after a second round of lithography, with the writing process being the same as described above. The second row is again marked by a red arrow. Scale bars equal 50 µm.

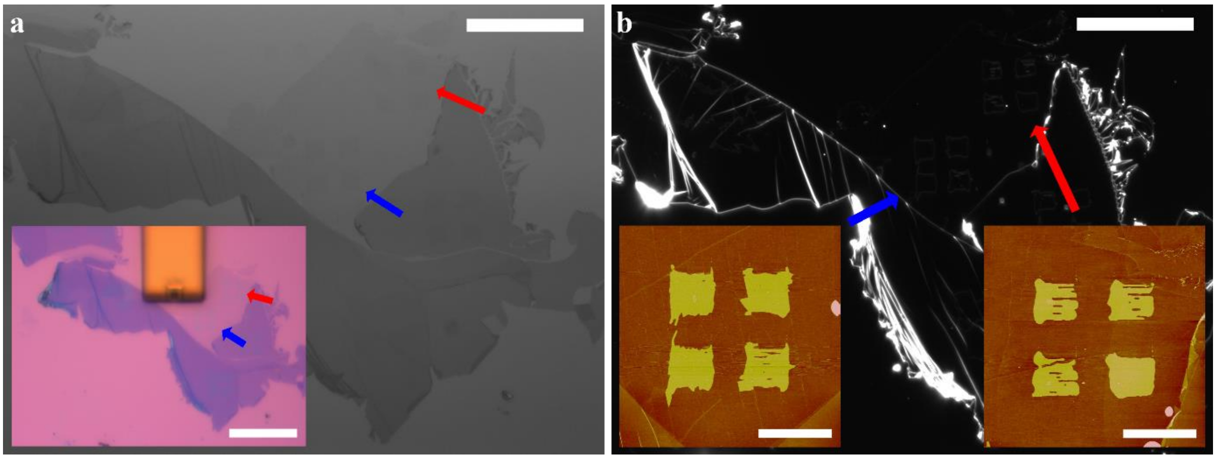

Supplementary Figure S2 | Lipid Patches written subsequently by single cantilevers. Bright field image (a) and dark field image (b) of a single layer graphene area after application of DOPC/DOPA patches (blue arrows) and pure DOPC patches (red arrows), scale bars equal 35 µm in main panels. The inset in (a) shows the area right after lithography with the cantilever loaded with the DOPC/DOPA mixture still hovering above the sample (scale bar equals 50 µm). The insets in (b) show corresponding AFM images of the different lipid patches (scale bars equal 10 µm).

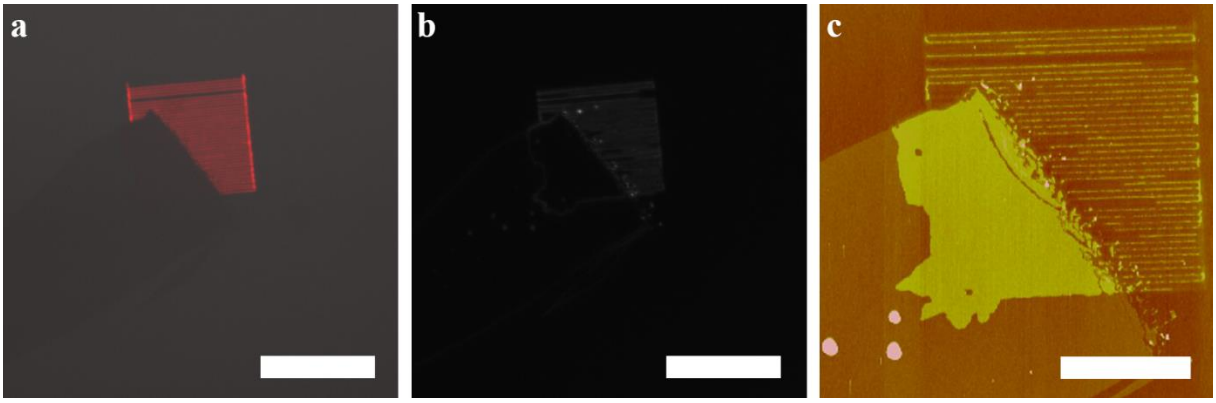

Supplementary Figure S3 | Lipid lines written with Rhod-PE mixture over the edge of a graphene sheet. (a) The combined bright field and flurescent image shows fluorescence quenching on the graphene. (b) The dark field image reveals a smooth membrane on the graphene in comparison to the still separated lipid lines on the silicon dioxide area. Scale bars in (a) and (b) equal 20 µm. (c) AFM image of the lipid structure clearly shows the distinct lines on the silicon dioxide (pitch 500 nm) while the lines on the graphene already merged to a smooth homogenous lipid membrane patch. To allow for the complete merging, enough material must be transferred from the tip to the substrate, therefore the still separate partly even discontinuous lines on the the silicon dioxide in contrast to the already merged and spreading membrane on the graphene indicates a significantly higher tip substrate transfer rate on the graphene in comparison to the silicon dioxide. Scale bar in (c) equals 10 µm.

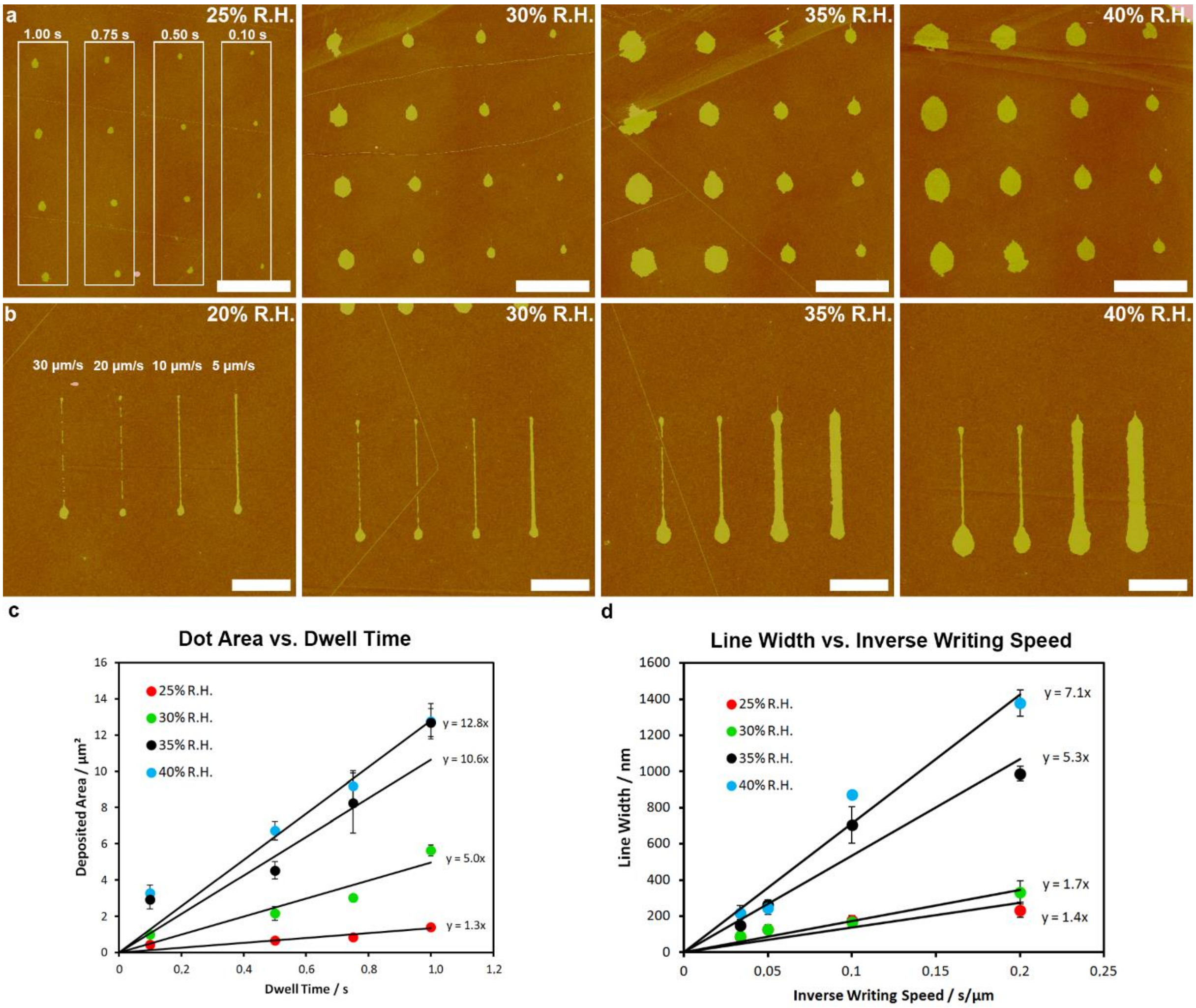

Supplementary Figure S4 | Study of lipid transfer in dot and line features dependence on dwell time and writing speed. (a) Dot patterns written with Rho-PE. The dots are spaced with a 10 µm pitch and each column is written with different dwell times as indicated in the first image. Scale bars equal 10 µm. (b) Line patterns of 10 µm lines with a pitch of 5 µm written at different speeds as indicated in the first image. Scale bars equal 5 µm. (c) Graph showing the data extracted from the images in (a), (d) graph showing the data extracted from the images in (b).

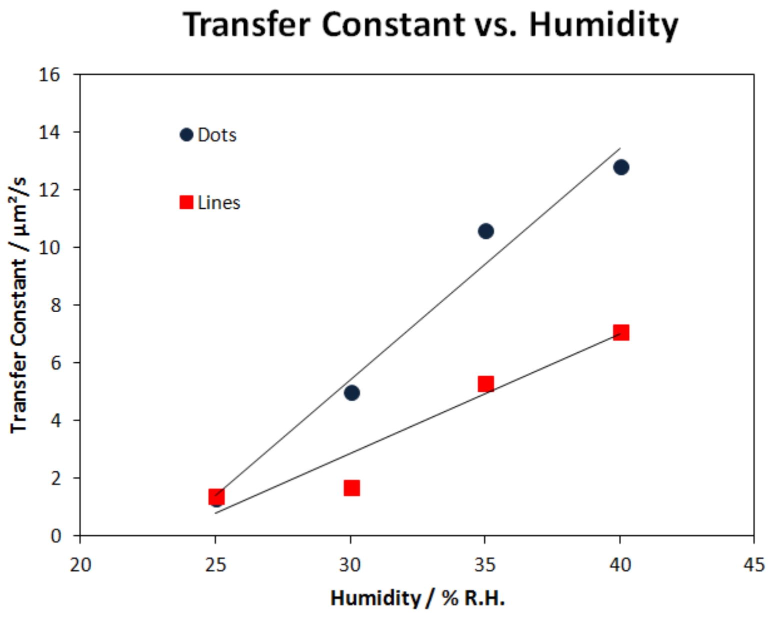

Supplementary Figure S5 | Dependence of the transfer constant on humidity for dot and line features. The graph shows the dependence of the transfer constant on humidity for dot patterns (blue circles) and line patterns (red squares).

Supplementary Table S1 | Transfer constants as derived from Supplementary Fig. S4c and d.

**Humidity / % R.H.**	**Transfer Constants / µm ^{2}/s**	
	**Dot Features**	**Line Features**
25	1.3	1.4
30	5.0	1.7
35	10.6 (11.6)*	5.3
40	12.8	7.1

* Excluding data point for 1 s dwell time. Value in brackets includes all data points.
